# Acceptability and Feasibility of an Evidence-Based Requisition for Bone Mineral Density Testing in Clinical Practice

**DOI:** 10.1155/2016/6967232

**Published:** 2016-12-06

**Authors:** Sarah E. P. Munce, Debra A. Butt, Rokeni (Sumi) Anantharajah, Susana Huang, Sonya Allin, Tarik Bereket, Susan B. Jaglal

**Affiliations:** ^1^Toronto Rehabilitation Institute, University Health Network, Toronto, ON, Canada; ^2^Department of Physical Therapy, University of Toronto, Toronto, ON, Canada; ^3^Department of Family and Community Medicine, University of Toronto, Research Department, Toronto, ON, Canada; ^4^Family and Community Medicine, The Scarborough Hospital, Scarborough, ON, Canada

## Abstract

*Introduction.* The purpose of this study is to understand the experience of primary care providers (PCPs) using an evidence-based requisition for bone mineral density (BMD) testing.* Methods.* A qualitative descriptive approach was adopted. Participants were given 3 BMD Recommended Use Requisitions (RUR) to use over a 2-month period. Twenty-six PCPs were interviewed before using the RUR. Those who had received at least one BMD report resulting from RUR use were then interviewed again. An inductive thematic analysis was performed.* Results.* We identified four themes in interview data: (1) positive and negative characteristics of the RUR, (2) facilitators and barriers for implementation, (3) impact of the RUR, and (4) requisition preference. Positive characteristics of the RUR related to both its content and format. Negative characteristics related to the increased amount of time needed to complete the form. Facilitators to implementation included electronic availability and organizational endorsement. Time constraints were identified as a barrier to implementation. Participants perceived that the RUR would promote appropriate referrals and the majority of participants preferred the RUR to their current requisition.* Conclusions.* Findings from this study provide support for the RUR as an acceptable point-of-care tool for PCPs to promote appropriate BMD testing.

## 1. Introduction

Clinical practice guidelines (CPGs) in Canada currently recommend bone mineral density (BMD) testing for patient fracture risk assessment [1, 2]. However, evidence of inappropriate testing has been identified in terms of over testing in low risk women [3, 4] and under testing in patients at high risk [4]. Furthermore, there is a need for better capture of risk information at the time of referral for BMD testing, not only to promote more appropriate BMD testing but also to facilitate accurate fracture risk assessment [5, 6]. Thus, primary care providers (PCPs) require accurate and consistent tools to communicate clinical criteria for referral. In fact, in a systematic review on the effectiveness of education strategies designed to change physician performance and health care outcomes, it was determined that systematic practice-based interventions, including reminders, were among the most effective strategies [7].

PCPs, including family physicians (FPs), currently order BMD tests using requisitions distributed by community facilities or hospital imaging departments; these have minimal, if any, reminders to include clinical information and patient specific risk factors relevant to fracture risk assessment. Based on recommendations of the Ontario BMD Working Group and in collaboration with the Ontario Osteoporosis Strategy [8], a “Recommended Use Requisition” (RUR) was developed to promote more appropriate BMD testing (see Supplemental File 1 for a copy of the RUR in Supplementary Material available online at http://dx.doi.org/10.1155/2016/6967232) (i.e., decrease testing among individuals at low risk of fracture and increase testing among individuals at high risk of fracture). The RUR has been designed to follow the diagnostic algorithm outlined in the 2010 Canadian Osteoporosis CPGs [1] for baseline testing and Choosing Wisely Canada's recommendations to reduce inappropriate test utilization, specifically utilization of repeat BMD tests (i.e., “don't repeat dual X-ray absorptiometry scans more often than every 2 years”) [9]. Recently, British Columbia, Manitoba, and Nova Scotia have developed standardized requisitions for BMD testing which communicate guidelines and act as point-of-care decision aids. Previous research by Rosenthal and colleagues (2006) [10] examined the influence of a computerized order entry system for radiology on ordering practices and demonstrated a decline in low utility examinations. However, to the best of our knowledge, there are no studies evaluating the impact of these requisitions on appropriate BMD testing or the user experience of these requisitions. Therefore, the purpose of this study was to understand the experience among PCPs of using the RUR for BMD testing in Ontario. Results from this study will be used to iterate the RUR, including the development of the RUR as an electronic decision aid (with a built-in system of reminders), with the ultimate aim of promoting more appropriate BMD testing in Ontario.

## 2. Methods

### 2.1. Design

We conducted semistructured interviews of PCPs in person and by telephone before and after using the RUR. A qualitative descriptive approach as described by Sandelowski [11, 12] informed the design, collection, and analysis of data.

### 2.2. Recruitment

Thirty PCPs who ordered BMD tests in their practices (all urban and rural community-based teaching sites affiliated with the University of Toronto) were invited to participate in this study between September and November of 2013. As such, a convenience sample was used. Recruitment ceased as analysis of the data (i.e., for baseline interviews and the second set of interviews) approached data saturation, which is defined as the point where successive interviews did not generate novel responses or themes [13]. This study received Research Ethics Board approval from the University of Toronto, Women's College Hospital, and the Scarborough Hospital. All participants provided informed consent.

### 2.3. Data Collection

Each participant took part in two one-on-one semistructured interviews lasting approximately 10–15 minutes each (i.e., before and after using the RUR). Two family medicine residents (Rokeni (Sumi) Anantharajah and Susana Huang) conducted the interviews which consisted of a combination of closed- and open-ended questions and were pilot-tested with other FPs. Probes were used to explore issues in greater depth and verify the interviewers' understanding of the information being collected [13]. The family medicine residents (Rokeni (Sumi) Anantharajah and Susana Huang) were trained by two members of the research team with experience in qualitative research methods (Sarah E. P. Munce and Tarik Bereket). All interviews were recorded by verbatim typed-note taking during the interviews by two members of the research team (Rokeni (Sumi) Anantharajah and Susana Huang) using password-protected laptops.

During the baseline interviews, information on the PCPs' demographics, current practices in ordering BMD tests (i.e., On average how many BMDs do you order in a month? In what situations do you feel inclined to order a BMD test? What “triggers” or “reminders” prompt you to order a BMD test? What risk factors do you consider when ordering a BMD test? What is the format in which BMD tests are ordered? What information do you include when you order a BMD test? Do you currently recommend to your patients a specific location to complete their BMD testing? If so, why or why not?), and initial impressions of the RUR were collected (i.e., What was your first impression of the RUR?). At baseline, participants were given three copies of the RUR to use over a two-month period. After one month, e-mail reminders were sent to participants encouraging use of the RURs. After 2 months, a second set of interviews was completed with participants who had received at least one BMD report resulting from their use of the RUR. This second interview focused on the ease and feasibility of using the RUR (i.e., Did you think the time required to complete the RUR was practical within the day-to-day running of your clinic? Why or why not? How did you find the overall flow of the RUR? What were the facilitating factors to implementing this form in your practice? What were the barriers to implementing this requisition in your practice?) and the perceived effectiveness of the RUR as a tool for osteoporosis screening and management (i.e., Did the BMD report appear any different as a result of using the RUR? If so, how? If yes, did the report change your management? Did you find the RUR allowed you to consistently communicate the patient risk factors for osteoporosis? Did you calculate your own fracture risk? If so, how did you do this? Did you agree with the risk factor assessment in the BMD report? Why or why not? Based on the information provided by the radiologist, do you know when to order your next BMD report? Based on your initial experiences with the RUR, did you prefer the RUR over your usual BMD requisition? Why or why not?)

### 2.4. Data Analysis

An inductive thematic analysis was performed on the data in order to explore the experience among PCPs of using the RUR for BMD testing [14]. We have used this approach to data analysis in prior related studies [6, 15].

## 3. Results

### 3.1. Description of Participants

Characteristics of the participating PCPs are presented in Table 1. Most participants practiced in urban or mixed urban-rural settings, with a roster size of 1000 to 1500 patients. Most PCPs (*n* = 16) stated that 50 to 75% of their practice population was older than 40, and many (*n* = 12) said 25 to 50% of their practice was older than 65. Twenty-six participants completed the initial interview and 15 completed the follow-up about ease of use. The following four themes were identified across all interview data: (1) positive and negative characteristics of the RUR, (2) facilitators and barriers for implementation, (3) impact of the RUR, and (4) requisition preference.

### 3.2. Positive and Negative Characteristics of the RUR

The majority of participants expressed positive characteristics of the RUR before (88%) and after use (93%) especially related to content and format. Participant 11 noted that “*first impression is good educational tool. Recommendations change all the time, due to new studies. This can help with the constant changes in terms of learning the new guidelines.”* Regarding the format, participant 18 stated that it is “*easy to follow. I like the fact that it is 3 boxes: baseline, follow-up or check any that applies, so it is easy to follow. I like the fact that it has some clarification at the bottom, so if you have any questions, [you] can refer there.*”

The majority of participants (93% or 14/15) indicated that completion of the RUR was practical for daily use. As one participant explained that* “the tick boxes are easy and there's not much to fill. The rest of the information is what we would typically fill out on any requisition with an area for the usual demographic sticker” *(Participant 15). The practicality of the RUR was also highlighted by Participant 4 as it is* “straightforward and provides a good reminder of the guideline.”*

All negative characteristics of the RUR related to the length of the form and the time required for its completion. Three participants indicated that it would be more time-consuming to complete the RUR versus the current forms because of the detailed content. Suggestions for changes to the requisition included incorporating the RUR into the regular diagnostic imaging requisitions currently in use (Participant 10), splitting the baseline section and the follow-up section into separate requisitions to make each resultant form shorter (Participants 12 and 20), and removing the section with an elaboration on risk factors as it might be unnecessary “*after reading a couple*” (Participants 11, 12, and 21).

### 3.3. Facilitators and Barriers for Implementation

The most commonly cited facilitator to implementation of the RUR was electronic availability (43% or 11/26 before use and 60% or 9/15 after use). Other frequently cited facilitators included standardization of the RUR (i.e., province-wide use of the RUR) and organizational endorsement (e.g., Choosing Wisely Canada). The most commonly cited implementation barrier was* “forgetting to use the RUR*.” Other implementation barriers included* “uncertainty about lab acceptance of the RUR*,*” “lack of hard copies*,*” “lack of staff familiarity*,*”* concerns about creating paper clutter with the implementation of a separate form for BMD referral, and* “time constraints*.*”*

### 3.4. Impact of the RUR

Before using the RUR, 19% (5/26) indicated that the RUR would positively impact patient management by FPs, as it “*might open the opportunity to screening the patients”* (Participant 18) and would provide “*reminders about risk factors, current practice guidelines and when to repeat BMD” *(Participant 5). After using the RUR, 53% (8/15) reported that it had changed their practice, serving as a* “good refresher”* of the guidelines, helping them “*identify high risk patients who require screening*.*”* Approximately one-quarter of the participants indicated that the RUR did not change their practice, but that it served as an educational tool. However, the participants underscored that this lack of change was attributable to the short-term implementation of the RUR and the fact that only 3 copies were provided to each participant. This meant there were “*too few to comment*” (Participant 14). Participant 21 also agreed that the RUR was “*not used often enough*.”

After using the RUR, all participants indicated that if the RUR became standardized in Ontario, it would “*decrease the number of inappropriate tests ordered and increase the number of appropriate tests ordered*” (Participant 20). One-third (5/15) of the participants indicated that the RUR could serve as a teaching tool: “*I can explain to people who don't need to get BMD using this form as it lists out the guideline clearly*” (Participant 14). Some participants (20%, 3/15) highlighted that the RUR led to “*covering more males getting BMD because they are usually forgotten, i.e. for the low weight males*” (Participant 7). Participants 7 and 20 also mentioned that the RUR could serve as a reminder for when to order follow-up BMD tests.

No participants perceived that the RUR had a negative impact on the resulting BMD reports. However, most (87%, 13/15) indicated that the RUR would have a positive impact on the communication between FPs and radiologists: “*I think it will increase the clinical correlation [between patient's clinical risk and BMD reported risk assessment] because of consistency of format”* (Participant 17). Participant 1 also stated, “*I find the patient is asked a lot of this information by the radiologist. This would save the radiologist time*.”

Three participants suggested changes to the RUR after use, to improve communication including adding a space for relevant medications (Participant 1) and a space for height, weight, or loss of weight (Participant 14).

### 3.5. Requisition Preference

Post-RUR use, 67% (10/15) of participants preferred the RUR to the current requisitions in use. As one participant explained “*this one is better than [the] current form, has the risk factors according to guideline. Is more comprehensive. Cut down on unnecessary BMDs*” (Participant 14). Similarly, Participant 13 stated that “*on one sheet of paper you have all the guidelines. You are not going to over use and under use. You won't have a patient try to book a scan and be turned away. It's good for patient compliance*.” Three participants (Participant 1, 6, and 10) preferred their current BMD requisition, citing the extra time needed to complete the RUR and the lack of organizational endorsement for the RUR: “*compared to the regular form, might take 30 s longer or 1 minute longer…probably use the form if endorsed*” (Participant 1).

## 4. Discussion

The usefulness of the RUR as a tool to remind PCPs of the CPGs, as identified in the current study, is consistent with our previous research that has demonstrated that FPs lack clarity about the 2010 Canadian CPGs on appropriate screening for osteoporosis as well as the intervals for BMD follow-up [15]. This may be due to the fact that there are multiple sources of recommendations for BMD testing which do not always agree with the 2010 guidelines [1]. For example, a recent survey identified 24 different sets of published clinical guidelines for BMD testing [16]. In Ontario, the situation is further confounded by the fact that the current physician fee schedule still refers to the 2002 Canadian CPGs [17] which do not emphasize fracture risk or provide clear guidance for referral of high fracture risk patients. Thus, the proposed RUR may hold the potential to be an effective point-of-care tool to remind PCPs of the CPGs, support clinical decision-making, and promote appropriate testing. At the same time, however, it is important to note that the current qualitative study about perceived effectiveness and acceptability of the RUR is just the first step towards this goal (i.e., towards creating an effective point-of-care tool) and that further study is warranted to understand how to create systems where physicians are more likely to make appropriate care decisions.

The PCPs in the current study also recognized the usefulness of the RUR as a tool to promote appropriate BMD testing. Evidence of inappropriate BMD testing has been previously documented [1–4], warranting the implementation of a tool such as the RUR. In Canada and the US, about 50% of women over 65 years of age and 81% of men have not had a BMD test [18–20]. In a systematic review of practice patterns in the management of osteoporosis after fragility fracture, BMD testing was performed in less than 15% of patients with recent fractures in 15 of 23 studies [19]. Most recently, in a sample of 2,025 BMD referrals (using the International Society for Clinical Densitometry guidelines), compared to those deemed appropriate, inappropriate referrals were more likely to have had less/missing information [21]. Thus, implementation of the RUR holds potential to ensure the consistent capture of clinical information necessary to make appropriate referrals. Relatedly, the PCPs in our study indicated that a potential impact of the RUR could be better communication of clinical risk factors to radiologists.

Many of the participating PCPs found that the format of the RUR was easy to follow. It is worth noting that these characteristics are in keeping with some of the valued principles of interaction design including an “aesthetic and minimalist design” (e.g., only relevant information is included) and a “match between system and the real world” (e.g., information appears in a natural and logical order) [22]. Despite this, some participants noted that a barrier to implementation was the amount of time needed to complete the RUR compared to current forms. Time pressure is a commonly cited barrier to the implementation of knowledge translation interventions [23, 24]. However, participants also identified electronic availability as a significant facilitator to implementation. Making the RUR available electronically could minimize the time required to complete the form by enabling autopopulation of fields and allow for a built-in system of reminders (e.g., to encourage PCPs to order more tests for individuals at high-risk of fracture). Moreover, this suggestion is in keeping with the increasing adoption of electronic medical records in primary care; the National Physician Survey (2014) [25] indicated that 79.4% of all respondents were planning to order diagnostic tests electronically in the next two years. Finally, PCPs also cited organizational support as a key facilitator to implementation. This is another commonly cited facilitator to the implementation of knowledge translation interventions/tools [23, 24]. Choosing Wisely Canada [9] is one potential venue that could provide organizational support and lend additional credibility to the RUR.

This study acknowledges some limitations. The context of this study is Ontario which has many more DXA machines and operators than other provinces. This context may limit the applicability of our findings. Study participants may have had more interest and knowledge about osteoporosis than the average FP, given that the participants were all from teaching sites affiliated with the University of Toronto. It is likely that the need for such a referral tool would be greater among a general population of FPs (i.e., among those less familiar with the CPGs). Further, the RUR was implemented for a short time period; more representative experiences of RUR use may occur with longer implementation periods.

Findings from this qualitative study provide support for the RUR as an acceptable point-of-care tool for PCPs for BMD testing. PCPs perceived that the RUR had the potential to improve appropriate BMD testing and the communication of risk factors between the PCP and the radiologist, which could ultimately improve the accuracy of BMD reports. However, future research must confirm this in order to ensure the successful provincial and national implementation of the RUR and consider how to create systems where physicians are more likely to make appropriate care decisions.

## Supplementary Material

The Recommended Use Requisition (RUR) was developed in collaboration with the Ontario Bone Mineral Density Working Group and the Ontario Osteoporosis Strategy. It is also based on the 2010 Canadian Osteoporosis clinical practice guidelines for baseline testing and Choosing Wisely Canada's recommendations to reduce inappropriate test utilization, specifically utilization of repeat BMD tests.

## Figures and Tables

**Table 1:** Characteristics of study participants.

Characteristic	RUR before use	RUR after use
	*N* = 26	*N* = 15
	*n* (%)	*n* (%)
*Gender*		
Female	16 (62)	10 (67)
*Age* (years)		
20–30	4 (15)	3 (20)
31–40	9 (35)	6 (40)
41–50	3 (12)	2 (13)
51–60	8 (31)	4 (27)
≥61	2 (8)	0 (0)
*Practice setting*		
Urban	18 (69)	13 (87)
Rural	5 (19)	1 (7)
Urban/rural	3 (12)	1 (7)
*Type of practice *		
Group	20 (77)	12 (80)
Family Health Team (FHT)	5 (19)	2 (13)
Solo	1 (4)	1 (7)
*Payment model*		
Family Health Organization	20 (77)	12 (80)
Family Health Group	3 (12)	3 (20)
Other (e.g., salary, FHT, NP)	1 (4)	0 (0)
Comprehensive Care Model	2 (8)	0 (0)
Fee-for-Service	0 (0)	0 (0)
*Size of practice or roster*		
<500	4 (15)	1 (7)
500–800	5 (19)	2 (13)
800–1000	4 (15)	3 (20)
1000–1500	12 (46)	8 (53)
>1500	1 (4)	1 (7)
*Resident*		
No	18 (69)	11 (73)
Yes	7 (27)	4 (27)
Nurse practitioner	1 (4)	0 (0)
*Time since residency* (range, years)	0–40	0–33
