# Supplementary material for: Acceptability and Feasibility of an Evidence-Based Requisition for Bone Mineral Density Testing in Clinical Practice
Source: J Osteoporos. 2016 Dec 6;2016:6967232. doi: 10.1155/2016/6967232 (PMC5168451; doi:10.1155/2016/6967232)
Supplement: Supplementary file 1 — The Recommended Use Requisition (RUR) was developed in collaboration with the Ontario Bone Mineral Density Working Group and the Ontario Osteoporosis Strategy. It is also based on the 2010 Canadian Osteoporosis clinical practice guidelines for baseline testing and Choosing Wisely Canada's recommendations to reduce inappropriate test utilization, specifically utilization of repeat BMD tests. [file 6967232.f1.docx]

**WCH HEADER HERE PATIENT ID HERE**

**BONE DENSITOMETRY REQUISITION (BMD)**

| **Referring Physician Name** (please print):____________________________________ **Signature**: ______________________  Date: _____/____/____ Billing number: ___________________ Tel: ___________________ Fax: ___________________  YYYY / MM / DD |
| --- |

- **BASELINE BMD**

| **Patients with any of the following risk factors (check ALL that apply):** | |
| --- | --- |
| - Female or Male age ≥ 65 - History of fragility fracture (after age 40)^1^ - Recent prolonged glucocorticoid use^2^ - Other high risk medication use^3^ | - Menopausal female (≥ 1 year post cessation of menstrual periods) with body weight < 60kg - Male age 50 – 64 with body weight < 60 kg |
| - Conditions associated with bone loss or fracture^4^ SPECIFY:______________________________________________ | |

- **FOLLOW UP BMD** Date of last BMD: _________ (YYYY/MM/DD)

| **For patients at LOW fracture risk on prior exam, OHIP will cover:**   - A second BMD test **3 YEARS AFTER** the baseline test - A successive BMD test (i.e. 3^rd^ or more) **5 YEARS AFTER** the last test |
| --- |
| **Follow up BMD tests at intervals of EVERY 2-3 YEARS are appropriate for most MODERATE or HIGH risk patients (including those recently discontinuing therapy).** ^5^  For any patient, follow up BMD Tests may be appropriate **AFTER 1 YEAR** if**:**   - Has a new fragility fracture^1^ - Active risk factor for bone loss^2,3,4^ - Significant bone loss on prior BMD exam^6^ - Initiated or changed to a new bone-sparing medication within the past year |
| **Comments:** |
| ^1^defined as fracture that occurs spontaneously such as vertebral fracture identified on X-ray or after minor trauma such as a fall from standing height or less, EXCLUDING craniofacial, hand, ankle and foot fractures  ^2^≥3 months in the prior year at a prednisone equivalent dose ≥ 7.5 mg daily  ^3^ e.g. aromatase inhibitors, androgen deprivation therapy, anticonvulsant therapy  ^4^e.g. primary hyperparathyroidism, osteogenesis imperfecta, uncontrolled hyperthyroidism, male hypogonadism, Cushing’s disease, chronic malnutrition or malabsorption syndrome, chronic liver disease, COPD, and inflammatory conditions (e.g. inflammatory bowel disease, lupus, rheumatoid arthritis)  ^5^refer to 2014 Choosing Wisely Canada recommendations: <http://www.choosingwiselycanada.org/recommendations/rheumatology/>  ^6^ OHIP defines significant bone loss as being in excess of 1% per year |
